# Colonization of the central nervous system as a key factor in the *Cryptococcus gattii* pathogenicity

**DOI:** 10.1080/21505594.2025.2594289

**Published:** 2025-11-24

**Authors:** Gustavo José Cota Freitas, Ludmila Gouveia-Eufrasio, Eluzia Castro Peres Emidio, Nalu Teixeira Aguiar Peres, Daniel Assis Santos

**Affiliations:** Departamento de microbiologia/Laboratório de Micologia, Universidade Federal de Minas Gerais, Belo Horizonte, Brazil

**Keywords:** *Cryptococcus gattii*, small cells, virulence, capsule, central nervous system, cryptococcosis

## Abstract

The *Cryptococcus gattii* species complex is one of the main etiological agents of cryptococcosis, a fungal infection that affects the lungs and progresses to meningoencephalitis. Neurological infections caused by this species are associated with a higher incidence of cryptococcoma, sequelae, and relapse. Despite its clinical relevance, the mechanisms that sustain the infection and persistence of *C. gattii* in the central nervous system (CNS) are still poorly understood. In this study, we performed a comprehensive phenotypic characterization of the virulence of five *C. gattii* strains, focusing on the CNS infectious biology. Significant differences in virulence among the strains were found, highlighting intracellular survival within macrophages and growth in the CNS as determining factors of disease severity. The relative capsule size between 1–2, as opposed to a marked increase (> 2), was associated with increased virulence, a phenomenon we term optimized capsule enlargement. Considering the high energetic cost of capsule synthesis, this pattern favors the fungal reproductive fitness and replication within the host’s tissues. These findings indicate that, in addition to the ability to reach the CNS, *C. gattii* needs to colonize it efficiently to cause severe clinical manifestations, with optimized capsule enlargement being a key factor for its survival in this microenvironment.

## Introduction

The *Cryptococcus gattii* species complex is among the main etiological agents of criptococcosis [1]. Currently, this complex includes five species (*C. gattii*, *Cryptococcus deuterogattii*, *C bacillisporus*, *Cryptococcus tetragattii* e *Cryptococcus decagattii*) [1–3]. This species emerged in 1999, during an outbreak in Vancouver Island and spread to the Pacific Northwest (PNW) of the United States [4–6]. Traditionally confined to tropical and subtropical regions, *C. gattii* demonstrated a broader ecological range given the outbreak in the temperate and climate of the Vancouver Island in 2006 [1].

Previously, we investigated the cellular and transcriptional remodeling dynamics of *C. neoformans* during infection, revealing an early-stage transcriptional and morphological changes that enhance reproductive fitness and tissue invasion [7]. In a later stage, changes that promote resistance to the immune response, such as the enlargement of the polysaccharide capsule, become critical for the progression of the disease [7]. However, this characterization is still limited in *C. gattii* strains, and the strategies used by this species during infection remains unclear. Furthermore, observing virulence phenotypes *in vitro* is insufficient to predict *in vivo* virulence, so the use of animal models combined to *in vitro* analysis enable the knowledge on several aspects of cryptococcal pathogenesis.

Cryptococcal infection typically begins in the lungs after the inhalation of fungal propagules and, in susceptible hosts, the fungus can disseminate to the central nervous system (CNS). For *C. neoformans*, the mechanisms of invasion and survival in the CNS are relatively well established, including transcellular, paracellular, and macrophage-Trojan horse passage across the blood–brain barrier (BBB), as well as inositol metabolism [8,9]. Current evidence suggests that *C. gattii* often displays greater persistence in the lungs and may be less efficient than *C. neoformans* in disseminating to the CNS although similar BBB-crossing mechanisms are likely presented [1,10–12]. Differences in ecological niches, host susceptibility, strain-specific adaptations in the CNS, and underdiagnosis of *C. gattii* infections may explain these differences between species [10,13]. Otherwise, CNS infections caused by the *C. gattii* complex are associated with a higher incidence of cerebral cryptococcomas, neurological sequelae, and relapses [1,14–17]. These clinical particularities highlight the importance of deepening our understanding of the infectious biology of *C. gattii* in the CNS. This knowledge gap is particularly relevant given that the transcriptional regulators involved in brain infection in *C. neoformans* are not functionally conserved within the *C. gattii* species complex [8].

In this study, we investigated the infectious biology of the *C. gattii* species complex at the early and late stages of infection. Early morphological changes play a key role in the establishment of infection, while resistance to the macrophages’ fungicidal activity may enhance translocation to the CNS through the “Trojan horse” mechanism. After dissemination, all strains demonstrated the ability to infect the CNS. However, only the most virulent strains were able to colonize the brain tissue and increase mice morbidity and mortality. At this stage, optimized increase of the polysaccharide capsule in the brain was revealed to be a critical factor for the virulence of *C. gattii*.

## Materials and methods

### Microorganisms and phenotypic characterization

The five *C. gattii* strains utilized in this study include R265 (clinical isolate, reference; American Type Culture Collection), along with four additional strains representing distinct genotypes/serotypes (WM779, WM178, WM179 and WM161) (Table 1). All strains were obtained from ATCC (Manassas, VA). Yeast cells were grown in Yeast Extract Peptone Dextrose (YPD—2% glucose, 2% peptone, and 1% yeast extract) or Minimal Medium (MM—15 mM glucose, 10 mM MgSO_4_ 7 H_2_O, 29.4 mM KH_2_PO_4_, 13 mM glycine, and 3 mM thiamine-HCl, pH 5.5) broth, for 48 h/72 h (exponential phase) at 37°C for each experiment. Growth rates were determined by spectrophotometry (OD 600 nm) for 72 h at 37°C. Each strain was tested in eight replicates, and growth was quantified by calculating the area under the curve (AUC) for comparative analysis.

**Table 1.** *C. gattii* complex strains used in this study.

Strain	Genotype/Serotype	Origin
WM 161 (ATCC® MYA-4562™)	VG III B	Environmental (Eucalyp.) – San Diego (USA)
WM 178 (ATCC® MYA-4561™)	VG II B	Clinical – Australia
WM 179 (ATCC® MYA-4560™)	VG I C	Clinical – Australia
WM 779 (ATCC® MYA-4563™)	VG IV C	Veterinary (Cheetah) – South Africa
R265 (ATCC® MYA-4093™)	VG II A	Clinical – Vancouver (Canada)

ATCC: American Type Culture Collection.

For morphological evaluation, cells were cultured in YPD and MM broth for 72 h at 37°C, then counterstained with India ink. Morphometric analyses were performed on a minimum of 100 cells using Image J software (http://imagej.nih.gov). The cell body diameter was measured excluding the capsule [7]. Capsule size was calculated by the ratio between the capsule thickness and the cell body radius. Total cell size was defined as the cell body diameter including the capsule. The surface/volume ratio was determined using the formula 3/r, where *r* = radius [18]. All phenotypic assays were performed in triplicate.

Melanin production was determined visually after cells were cultured in solid minimal medium supplemented with 1 mM l-dopa (Sigma Aldrich) and incubated for five days at 37°C. Quantification of laccase and urease enzymatic activity was determined as previously described [7,19–21]. Briefly, to evaluate laccase activity, *C. gattii* samples were grown in solid minimal medium (15 mM glucose, 10 mM MgSO4, 29.4 mM KHPO4, 13 mM glycine, and 18 g/L bacteriological agar, pH 5.5) for 72 hours and then 1 × 10^8^ viable cells/mL were suspended in PBS. Then, 100 µL of 10 mM ABTS (2,2’-azinobis(3-ethylbenzthiazoline-6-sulfonate) was added to 900 µL of the fungal suspension and incubated overnight. After incubation, the absorbance of the supernatant was read at 420 nm. The absorbance detected corresponds to the oxidation of ABTS by the laccase enzyme. Thus, the value obtained was indirectly proportional to the enzyme activity [22].

To determine urease activity, 1x10^8^ viable cells/mL, previously cultured in YEPG (Yeast Extract-Glucose-Peptone agar) medium (3 g/L yeast extract, 5 g/L peptone, 5 g/L glucose and 18 g/L agar), were added to RUH (Rapid Urea Broth) broth (2.0 g urea, 0.01 g yeast extract, 1.0 mg phenol red, 0.1365 g KH2PO4 and 0.1425 g Na2HPO4; in 100 mL of distilled water) and incubated at 37°C for 4 hours of incubation [19]. Then, the solution was centrifuged and the supernatant removed for later spectrophotometric reading with absorbance at 560 nm [19].

### In vivo assays

Male C57/BL6 mice (*n* = 6) with approximately 4–6 weeks of age (from the Universidade Federal de Minas Gerais, Brazil) were used to determine mortality curves, fungal burden, and morphological kinetics after infection with *C. gattii*. Animals were anesthetized with ketamine (10 mg/kg) and xylazine (4 mg/kg) and inoculated intratracheally with a fungal suspension of 1 × 10^5^ yeast cells in a final volume of 30 μl PBS. Animals were monitored daily for survival analysis and behavioral assessment using the SmithKline/Harwell/Imperial College/Royal Hospital/Phenotype Assessment (SHIRPA) protocol. This protocol provides reliable information on murine brain dysfunction and general status. Individual parameters assessed were grouped into five functional categories: neuropsychiatric status, motor behavior, autonomic function, muscle tone and strength, and reflex and sensory function. The score for each category was calculated as previously described [23]. Table S1 describes the parameters analyzed.

Alternatively, groups of five mice were infected as described above with each strain used in this work. At this stage, to compare the morphological kinetics of *C. gattii* and *C. neoformans*, animals were also infected with *C. neoformans* strains (WM626, WM628, WM629 and WM148) (Table 2) with distinct virulence patterns, as we previously described [7]. Each group received a specific strain. After 6 h, 24 h and 240 h of infection, the animals infected with each strain were euthanized and the bronchoalveolar lavage, lungs, and brain were excised and homogenized. The material was plated on YPD agar medium to determine the colony forming units (CFU), as previously described [7]. The specimens were also used for morphological analysis of *C. gattii*. After homogenization, they were fixed on a slide with India ink, followed by visualization under an optical microscope and image capture. The cell and capsule sizes of at least 100 yeasts from each condition were measured using the Image J program (http://rsb.info.nih.gov/ij/ (accessed 4 January 2021); National Institutes of Health, NIH, Bethesda, MD, USA) [24]. During the statistical analysis, the results were grouped according to species, allowing us to identify a morphological profile and evaluate the possible differences between *C. gattii* and *C. neoformans*.

**Table 2.** *C. neoformans* complex strains used in *in vivo* assay.

Strain	Genotype/Serotype	Origin
WM 148 (ATCC® MYA-4564™)	VN I A	Clinical – Australia
WM 626 (ATCC® MYA-4565™)	VN II A	Clinical – Australia
WM 628 (ATCC® MYA-4566™)	VN III AD	Clinical – Australia
WM 629 (ATCC® MYA-4567™)	VN IV D	Clinical – Australia
H99 (ATCC® 208,821™)	VNI – A	Clinical – New York

The use of animals in this study was approved by the Ethics Committees for Animal Use of the Federal University of Minas Gerais (CEUA protocol 235/2017). The determination of the sample size was based on the Cochran model. The animals were kept in groups of up to 4 individuals, in cages with food and water ad libitum. The animals were kept following the standards of the National Council for the Control of Animal Experimentation (CONCEA) and the Brazilian College of Animal Experimentation (COBEA). During the experiments, mice were humanely euthanized with an intraperitoneal injection of 100 µL of a 10:1 (mg:mg) ketamine: xylazine solution, following the 2020 AVMA (American Veterinary Medical Association) Euthanasia Guidelines.

### Macrophage assays

Bone marrow-derived macrophages (BMDM) were used to assess the susceptibility of *C. gattii* strains to the macrophage fungicidal activity. The assay was performed as previously described [7,25]. Briefly, bone marrow cells were collected from the tibias and femurs of male C57BL/6 mice. The cells were then cultured in differentiation medium for 1 week at 37°C/5% CO_2_ [26]. Adherent cells differentiated into macrophages were resuspended with RPMI medium and transferred to a sterile polypropylene tube. Cell viability was determined with Trypan Blue (Sigma-Aldrich, Burlington, MA, USA), followed by plating in 24-well plates to determine the percentage of phagocytosis (PP) and fungicidal activity (FA), or in 96-well plates for reactive oxygen species (ROS) and peroxynitrite (PRN) quantification, as previously described [7,27].

PP and killing assays were assessed at 3- and 24-hours post-infection of BMDMs. For PP analysis, glass coverslips were placed in 24-well plates, and BMDMs were seeded and infected at a yeast-to-macrophage ratio of 5:1. At the indicated time points (3 h and 24 h), coverslips were retrieved and stained using Panotico Rápido (Laborclin, Pinhais, Paraná, Brazil). The phagocytosis rate was calculated by determining the percentage of macrophages containing internalized yeast cells under light microscopy. For the killing assay, supernatants were removed, and extracellular yeasts (both non-internalized and non-adherent) were eliminated by two PBS washes. Subsequently, macrophages were lysed with 200 μL of sterile distilled water for 30 minutes at 37 °C. Lysates were plated on YPD agar, incubated, and colony-forming units (CFU) were counted and reported as CFU/mL. All assays were conducted with six technical replicates, and the data reflect three independent experimental repeats [7,28].

ROS and PRN levels were quantified using 2,7-dichlorofluorescein diacetate (DCFH-DA) and dihydrorhodamine 123 (DHR 123), respectively – both obtained from Invitrogen (Thermo Fisher Scientific, Waltham, MA, USA). Infected BMDMs were incubated with the respective probes for 3 and 24 hours at 37 °C. Following incubation, fluorescence was recorded using excitation at 485 nm and emission at 530 nm [29]. Results were expressed as arbitrary fluorescence units (AU) ± standard error (SE) [7,23,30,31], representing intracellular ROS and PRN levels of the fungus and macrophages [7].

### Fungal burden kinetics and brain histology after intracranial infection

A 1 × 10^2^ CFU/5 µL inoculum was prepared and quantified using a Neubauer chamber with Trypan Blue. The animals were anesthetized, immobilized, and infected intracranially using a 30‑gauge needle attached to an insulin syringe, equipped with a stopper to limit penetration >1 mm. The injection site was on the cranial midline, located 6 mm posterior to the orbit, where the inoculum was delivered [32]. Each group of nine animals: five for fungal burden quantification and four for histological examination.

At 6 h, 24 h, and 240 h post-infection, animals were euthanized, and their brains were harvested. The tissue was weighed and homogenized in 1 mL of sterile PBS. Aliquots were plated on YPD agar and incubated at 37 °C for 48 h, with colony counts expressed as CFU/g of tissue. For histological detection of melanin, brain samples were fixed in formalin, embedded in paraffin, and stained using the Fontana–Masson method [33]. After microscopic analysis, the presence of melanin was considered as brown to black coloration in the yeasts.

### Statistical analysis and preliminary data

All statistical analyses were performed using GraphPad Prism, version 5.00 for Windows (GraphPad Software, San Diego, CA, USA). Results with *p* < 0.05 were considered statistically significant. Survival curves were evaluated using the *log-rank* test. For behavioral parameters, the area under the curve (AUC) analysis was applied. Laccase, urease, morphology, phagocytosis, IPR assay, ROS and PRN measurements, and CFU counts were analyzed using analysis of variance (ANOVA), followed by Tukey’s multiple comparison test.

Some of the preliminary data presented in this manuscript were part of the doctoral thesis entitled: “A biologia de *Cryptococcus* spp.: do remodelamento celular e transcricional ao tratamento.” The thesis was cited in Figures 2 and 3, where some preliminary data used are shown.

## Results

### C. gattii strains show distinct growth rates and morphological characteristics in vitro

**Figure 1.**
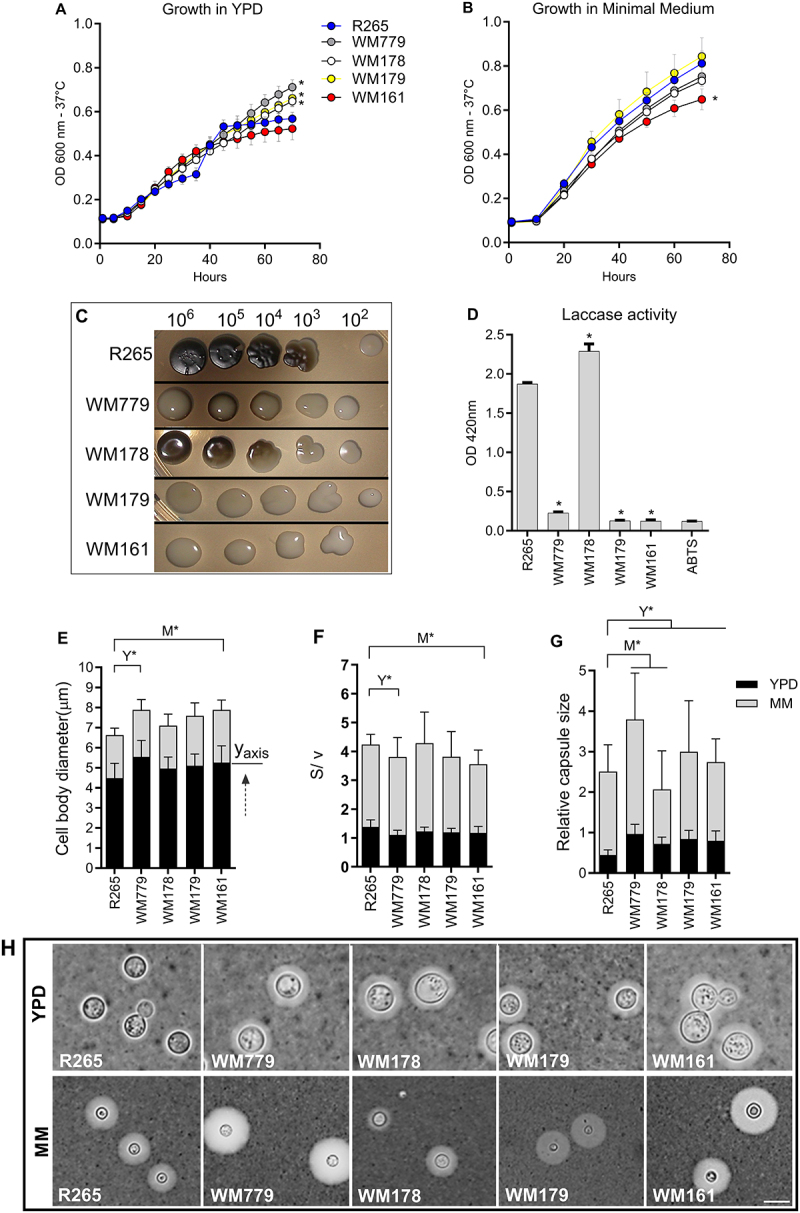
Phenotypic analysis of *C. gattii* strains *in vitro*. A) Growth curves of each strain in Yeast Extract–Peptone–Dextrose (YPD) broth at 37°C. B) Growth curves of each strain in Minimal Medium (MM) broth at 37°C. A-B) Graphs represent mean ± sd and are representative of 3 independent experiments. Significance was determined using analysis of the area under the curve (auc). Mean area values were compared with R265 (*p < 0.05). C) Visual analysis of melanin production after fungal growth in solid minimal medium with L-DOPA at 37°C for 7 days. (D) Laccase activity measured in mm (ABTS: 2,2’-azinobis(3-ethylbenzthiazoline-6-sulfonate) after 7 days at 37°C. E) Cell body diameter in YPD or in mm at 37°C after 72 h. Dashed arrow: indicates that the MM y-axis starts at the end of each YPD bar. F) Surface/volume ratio in YPD or in MM at 37° after 72 h. G) Relative capsule size in mm at 37 °C. C-G) Graphs represent mean ± SD and are representative of 3 independent experiments. Significance was determined using analysis of variance (anova), followed by Tukey’s multiple comparison test. Mean values were compared with R265 (*p < 0.05; M* Strain analysis on mm; Y*= Strain analysis on YPD). (H) India ink counter-staining of *C. gattii* after growth in mm for 72 hours at 37 °C. Scale bar: 5 μm.

Initially, general aspects of the morphophysiology of five *C. gattii* strains were analyzed, representing each genotypes/serotypes of this species complex. Growth curves of the five isolates at 37°C in Yeast Extract Peptone Dextrose (YPD) broth revealed a significantly higher growth rate for the strains WM779 (*p* < 0.0001), WM178 (*p* < 0.0001), and WM179 (*p* < 0.0001) compared to the reference strain R265 (Figure 1(A)). In minimal medium (MM) broth, only WM161 (*p* < 0.0001) strain showed reduced growth compared to R265 (Figure 1(B)). Melanin production was lower in strains WM779, WM161, and WM179 compared to R265 (Figure 1(C)), which was confirmed by the low laccase activity in these strains (WM779: *p* < 0.0001; WM161: *p* < 0.0001; WM179: *p* < 0.0001) compared to R265 (Figure 1(D)). By analyzing the cell body diameter and the surface-to-volume (S/V) ratio, the WM779 strain exhibited a larger cell body (*p* < 0.0001), and a lower S/V ratio (*p* < 0.0001) in YPD compared to the reference strain R265 (Figures 1(E,F)). A similar profile was observed for the strain WM161 (*p* = 0.0008; *p* < 0.0001 respectively) compared to R265 after culture in MM (Figures 1(E,F)). Compared to the reference strain R265, all strains had relatively larger capsules (*p* < 0.0001) in YPD (Figure 1G). In MM, strain WM779 (*p* = 0.001) exhibited the largest capsule size, while WM178 (*p* = 0.0003) had a smaller capsule compared to R265 (Figure 1(G,H)).

### C. gattii strains cause different degrees of disease severity in mice

After investigating the *in vitro* virulence’s attributes of the *C. gattii* strains, we evaluated whether those factors could predict *in vivo* virulence. Mice were infected intratracheally, and the survival, behavioral changes, and fungal burden were evaluated (*n* = 6).

**Figure 2.**
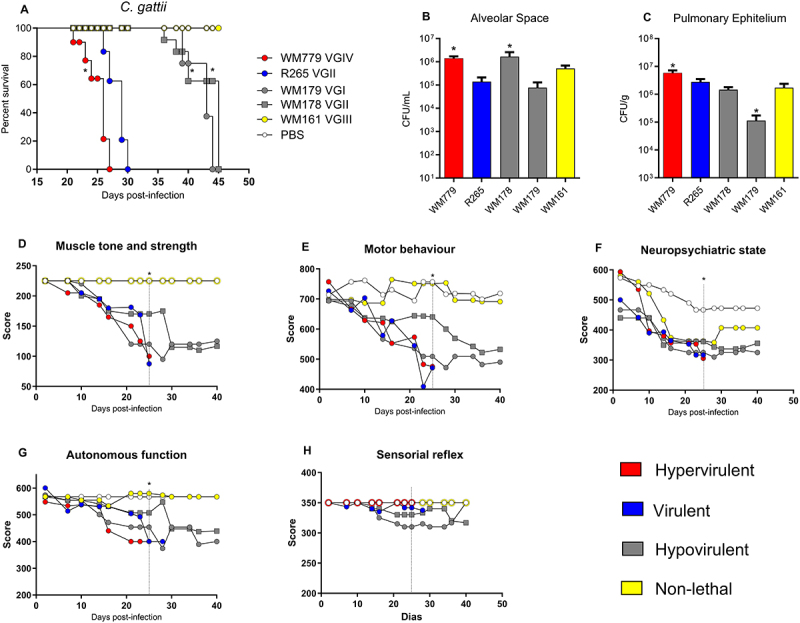
Disease severity profile caused by *C. gattii* strains in experimental cryptococcosis. (A) Survival curve of mice after intratracheal infection with 1x10^5^ *C. gattii* cells. Survival curves were generated from the results obtained with 6 mice per strain and evaluated for statistical significance with Kaplan-Meier survival curves, and *p* values were obtained from a log-rank test, *p < 0.05 compared with R265. This data was presented in Gustavo Freitas’ Ph.D thesis [34]. (B–C) Fungal burden in the alveolar space (AS) and pulmonary epithelium (PE) 10 days post infection. Graphs represent mean ± SD and are representative of 3 independent experiments with 5 mice per group. Significance was determined using analysis of variance (anova), followed by Tukey’s multiple comparison test. Mean values were compared with R265 (*p < 0.05). (D–H). Behavior analysis after infection with *C. gattii* strains. Significance was determined using analysis of variance (ANOVA), followed by Tukey’s multiple comparison test. Dashed lines indicate the statistical difference of hypovirulent and non-lethal strains compared with R265 (*p < 0.05) observed on day 25 post-infection. Virulence was classified according to the mean lethality time (MLt), with hypervirulent (red) range = 23–27 days, virulent (blue) range = 26–30 days, hypovirulent (gray) range = 36–45 days and non-lethal (yellow) range of MLt =  > 100 days.

Differences in the lethality were found for all strains (Figure 2(A)). Strain R265 led to lethality between 27 and 31 days (Lethality of 50% - Lt_50_ = 29 days) (Figure 2A). WM779 (*p* = 0.006) caused an earlier lethality (Lethality of 50% - Lt50 = 24 days), while the strains WM178 (Lt50 = 40 days) (*p* < 0.0001) and WM179 (Lt50 = 41 days) (*p* < 0.0001) led to longer survival compared to R265 (Figure 2(A)). The strain WM161 was not able to cause lethality during the experimentation protocol (Non-lethal – NL), but it induced behavioral changes at the beginning of the infection (Figure 2(A),(D–H)). According to survival, the strains were classified as hypervirulent (Lethality range/min – max/- Lt_r_ = 23–27 days), virulent (Lt_r_ = 26–30 days), hypovirulent (Lt_r_ = 36–45 days) and non-lethal (> 100 days) (Figure 2(A)).

The fungal burden recovered from bronchoalveolar space and pulmonary epithelium was significantly higher for the hypervirulent strain WM779 (*p* < 0.0001; *p* = 0.0018, respectively) compared to R265 (virulent) (Figure 2(B,C)). The WM178 strain, although hypovirulent, also presented a high fungal burden in the alveolar space. However, only the WM179 strain presented a significantly reduced fungal burden in the lung epithelium.

Although the fungal burden in the lung epithelium and alveolar space was not directly related to mice mortality, and thus, virulence; the hypervirulent (WM779) and virulent (R265) strains presented greater morbidity, as observed in all behavioral parameters evaluated, except for the sensory reflex (Figure 2(D–H)). The hypovirulent strains (WM178 *p* < 0,0001; WM179 *p* < 0.0001) presented a less intense behavioral impact compared to R265 at the 25th day after infection.

### The in vivo morphological kinetics of C. gattii revealed the presence of small cells in the early stages of infection and later capsule enlargement, independent of the lethality profile

Considering the *in vitro* morphological variation of *Cryptococcus* spp., we then evaluated how these changes occur during lung infection and how they are related to virulence. At this stage, in line with international animal welfare guidelines (such as the 3Rs principles: Reduction, Refinement and Replacement), we chose to restrict the number of strains tested in order to minimize the number of animals used, without compromising the quality of the data obtained. Mice (*n* = 5) were infected with four strains (WM779, R265, WM179, and WM161) representative of each virulence phenotype and euthanized after 6, 24 or 240 hours after infection. Subsequently, the morphology was determined directly from the pulmonary epithelium (PE) and alveolar space (AS). Morphology was not evaluated in the central nervous system due to technical problems in visualizing yeasts in the brain homogenate, given the decreased fungal burden in this organ due to dissemination after intratracheal infection.

**Figure 3.**
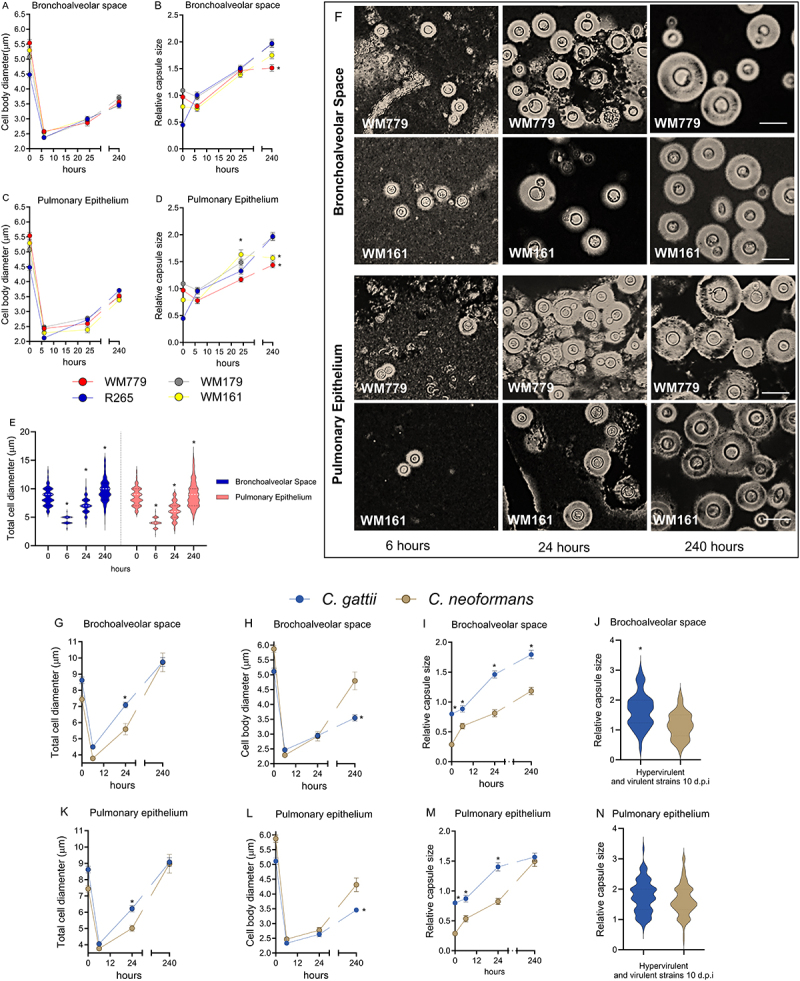
Dynamics of morphological variation of *C. gattii in vivo* and comparative analysis with *C. neoformans*. A-D) Morphological variation in the alveolar space (AS) and pulmonary epithelium (PE) of the four analyzed *C. gattii* strains after 6, 24 and 240 hours of intratracheal infection. Graphs represent mean ± SD and are representative of 3 independent experiments with 5 mice per group. Significance was determined using analysis of variance (ANOVA), followed by Tukey’s multiple comparison test. Mean values were compared with R265 (* *p* < 0.05). E) Total cell diameter in AS and PE for all *C. gattii* strains after 0 and 240 hours of infection. Mean values were compared with 0 hour of infection (*p < 0.05). F) India ink counter-staining of the yeasts recovered from infected mice. Scale bar:10 μm. (A-F) These data were presented in Gustavo Freitas’ Ph.D thesis [34]. G-N) Morphological variation in alveolar space (as) and pulmonary epithelium (pe) of four strains of *C. gattii* and four strains of *C. neoformans* after 6, 24 and 240 hours of intratracheal infection – each group was infected with a strain. Mean values were compared with *C. neoformans* (*p < 0.05) at each time of infection.

After 6 hours of infection, all strains showed a small cell body in the alveolar space and pulmonary epithelium, compared to the initial inoculum, leading to a predominance of small cells (≤3 µm) (Figure 3(A–D)). This data points to an early fungal replication right after reaching the host tissues. At this early time point of infection, there was an increase in the capsule-to-cell ratio for strain R265 (Figure 3(B,D)). After 24 hours, strain WM161 (*p* = 0.008) showed a larger relative capsule size compared to R265. At this time-point, tissue compartmentalization was also observed, with a higher percentage of small cells in the lung epithelium compared to the alveolar space (Table 3), indicating the greater ability of smaller cells to cross membranes and invade tissues.

**Table 3.** *C. gattii* cell body diameter variation in vivo.

Cell body diameter (µm)	Cells (%)							
	Bronchoalveolar Space				Pulmonary Epithelium			
	6 hours				6 hours			
	WM779	R265	WM179	WM161	WM779	R265	WM179	WM161
≤3	100	100	100	100	100	100	100	100
4 – 5	0	0	0	0	0	0	0	0
6 – 9	0	0	0	0	0	0	0	0
>10	0	0	0	0	0	0	0	0
	24hours				24hours			
≤3	83.3	79.6	84.21	83.3	98.1	96.02	95.02	100
4 – 5	16.7	20.4	15.79	16.7	1.9	3.92	4.98	0
6 – 9	0	0	0	0	0	0	0	0
>10	0	0	0	0	0	0	0	0
	240 hours (10 days)				240 hours (10 days)			
≤3	62.7	49.05	41.2	40.1	49.06	33.3	50.0	60.7
4 – 5	35.0	49.0	58.8	59.9	49.01	62.7	50.0	39.3
6 – 9	2.5	1.95	0	0	1.93	4.0	0	0
>10	0	0	0	0	0	0	0	0

After 10 days of infection, strains WM779 (*p* < 0.0001) and WM161 showed significantly smaller capsules in the alveolar space (Figure 3(B)) (*p* < 0.0001; *p* = 0.006; respectively) and in the lung (*p* < 0.0001; *p* = 0.002; respectively) (Figure 3(D)) compared to R265. Interestingly, after 6 (*p* < 00001) and 24 (*p* < 0,0001) hours of infection, fungal cells presented a significantly smaller total size compared to the initial inoculum (time 0) in the alveolar space and lung epithelium. However, in the late stage of infection, they returned to their original size (capsule + cell body), but with a larger capsule-to-cell body ratio at both sites (Figure 3(E,F)). However, no correlation was observed between virulence and cell size or capsule enlargement.

### The morphological kinetics of C. gattii diverges from C. neoformans in all stages of infection

Based on the average morphological measurements of the different lineages, we compared the morphological kinetics of *C. gattii* species complex and *C. neoformans* (WM626, WM628, WM629, and WM 148) strains in the alveolar space and pulmonary epithelium at different stages of infection (Figure 3(G–N)). At 6- and 24-hours post-infection, both species complexes showed reductions in total cell size and cell body diameter compared to the initial inoculum (time 0)(Figure 3(G,K)). However, at 24 hours post-infection, *C. gattii* had a significantly larger total cell diameter than *C. neoformans* in both AS (*p* = 0.003) and PE (*p* = 0.008) (Figure 3(G,K)), although this difference was not maintained at day 10. In contrast, cell body size remained similar between complexes throughout the infection, except at day 10, when *C. gattii* had a significantly smaller cell body than *C. neoformans* in the AS (*p* = 0.004) and PE (*p* = 0.002) (Figure 3(H,L)).

On the other hand, *C. gattii* displayed a significantly larger relative capsule size at all stages of infection in the alveolar space (Figure 3(I)). A similar pattern was observed in the pulmonary epithelium, except at 10 days post-infection, when the relative capsule size became similar between the complexes (Figure 3(M)).

As demonstrated in a previous study, the capsule size of *C. neoformans* can vary according to the strain’s virulence profile [7]. Based on this, we performed a comparative analysis between both species complexes, considering only the strains classified as virulent and hypervirulent. In this analysis, we observed that the relative capsule size of *C. gattii* remained significantly (*p* < 0.0001) larger in the alveolar space after 10 days of infection compared to *C. neoformans*, a pattern not identified in the lung epithelium (Figure 3(J,N)).

Despite the increase in the polysaccharide capsule in the alveolar space and pulmonary epithelium, this dynamic change was not sufficient to explain the differences in virulence for *C. gattii* strains, in contrast to what was observed for *C. neoformans*. On this basis, we investigated other parameters that might contribute to the better understanding of *C. gattii* virulence determinants, including intracellular survival, capsule size in the brain and ability for CNS invasion and colonization.

### Resistance to fungicidal activity of macrophages, optimized expansion of the polysaccharide capsule and fungal proliferation in the central nervous system (CNS) are determinants for C gattii virulence

**Figure 4.**
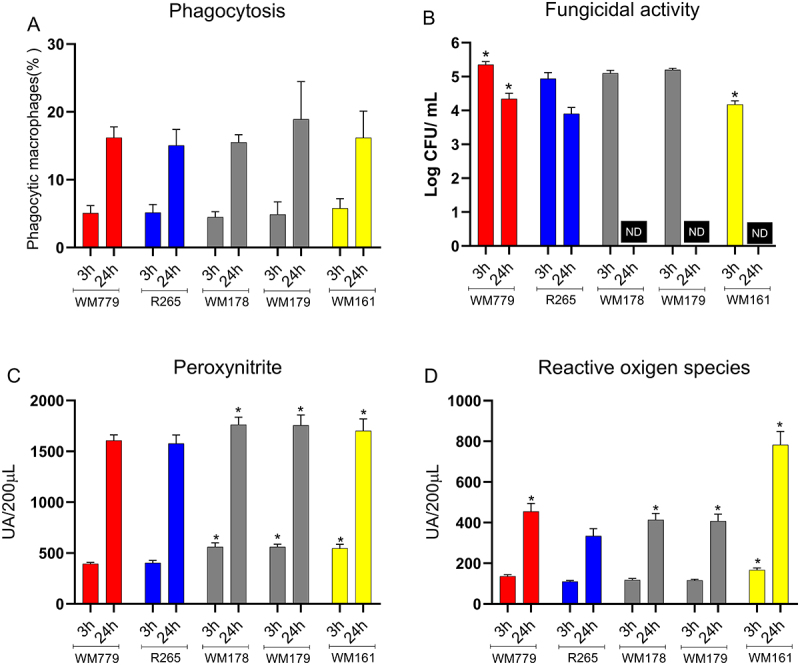
*Cryptococcus gattii* strains susceptibility to phagocytosis and fungicidal activity of macrophages. (A) Phagocytic index after 3 h and 24 h of infection. (B) Colony forming units (CFU) determination represents viable yeast cells internalized by macrophages in each time point. (C) peroxynitrite (PRN) and (D) Reactive Oxygen Species (ROS) production after 3 and 24 hours of infection by *C. gattii*. Graphs represent mean ± SD and are representative of 3 independent experiments. Significance was determined using analysis of variance (ANOVA), followed by Tukey’s multiple comparison test. Mean values were compared with R265 (*p < 0.05). au: arbitrary units of fluorescence. ND: Not detected.

Considering the importance of intracellular survival of *C. gattii* during infection, the interaction between the fungus and macrophages was evaluated. The phagocytosis rate did not vary significantly among the strains (Figure 4(A)), but the hypervirulent strain WM779 exhibited greater proliferation inside macrophages at 3 and 24 hours when compared to R265 (Figure 4(B)) (*p* < 0.0001; *p* = 0.0002; respectively). The hypovirulent strains exhibited proliferation similar to R265 in the first 3 hours, while the non-lethal strain WM161 (*p* = 0.0048) had lower survival (Figure 4(B)). On the other hand, after 24 hours of infection, both the hypovirulent and the non-lethal strains were no longer detected (Figure 4(B)).

Macrophages infected with the hypervirulent and virulent strains produced less peroxynitrite (PRN) after 3 and 24 hours of infection compared to R265 (Figure 4(C)). After 24 hours, ROS levels were higher for WM779 and hypovirulent strains compared to R265 (*p* < 0.0001) (Figure 4(D)). On the other hand, macrophages infected with the non-lethal strain showed a significant (*p* < 0.0001) increase in ROS and PRN compared to R265, at both time points (Figure 4(C,D)).

**Figure 5.**
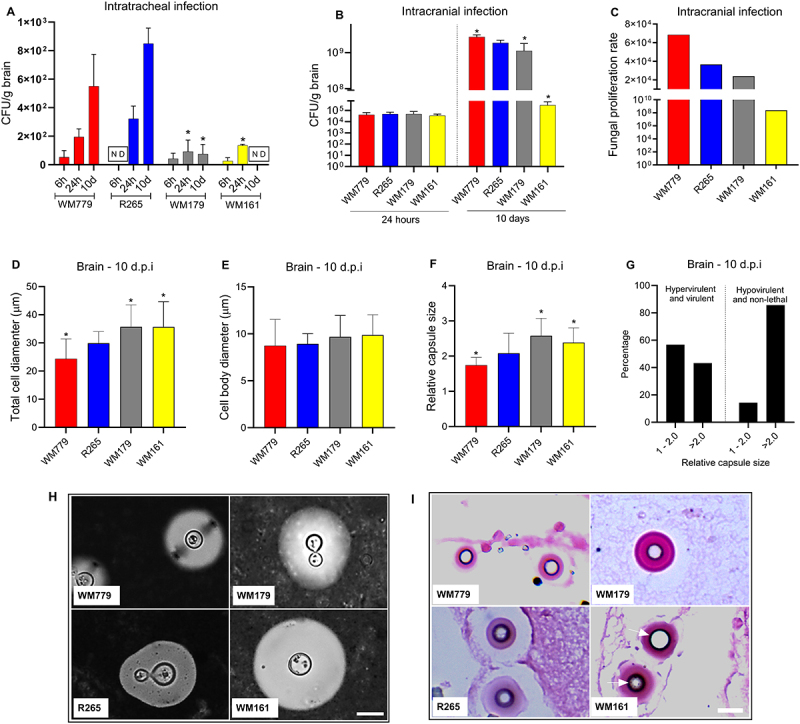
Virulence profile of *C. gattii* strains in the brain. A) Fungal burden in the brain after 6 h, 24 h, and 10 days of intratracheal infection. B) Fungal burden in the brain after 24 h, and 10 days of intracranial infection. C) Fungal proliferation rate in the brain after 10 days of intracranial infection. D) Total cell diameter in the brain 10 days post intracranial infection (10 d.P.i). (E) Cell body diameter in the brain 10 d.p.i. F) Relative capsule size in the brain 10 d.p.i. Graphs represent mean ± SD and are representative of 3 independent experiments. Significance was determined using analysis of variance (anova), followed by Tukey’s multiple comparison test. Mean values were compared with R265 (*p < 0.05). G) Percentage relative capsule size in the brain 10 d.p.i. according to virulence. (H) India ink counter-staining of *C. gattii* in the brain 10 days after intracranial infection. Scale bar: 10 μm. I) Brain stained with Fontana–Masson after 10 days of intracranial infection. White arrow indicates the stained fungus due to the presence of melanin in the cell wall. Scale bar: 50 μm.

Since *C. gattii* is an important pathogen associated with meningoencephalitis, the kinetics of fungal burden in the brain after intratracheal and intracranial infection was evaluated with the representative strains of each lethality phenotype. All strains were able to reach the CNS after 6 or 24 hours of intratracheal infection (Figure 5(A)). Interestingly, the hypervirulent and virulent strains were able to increase the fungal burden over time, while the less virulent strains were not able to grow in the CNS, with a lower fungal burden (compared to R265) after intratracheal infection (Figure 5(A)) (*p* < 0.05). The non-lethal strain WM161 was not detected in the CNS after 10 days of intratracheal infection (Figure 5A).

**Figure 6.**
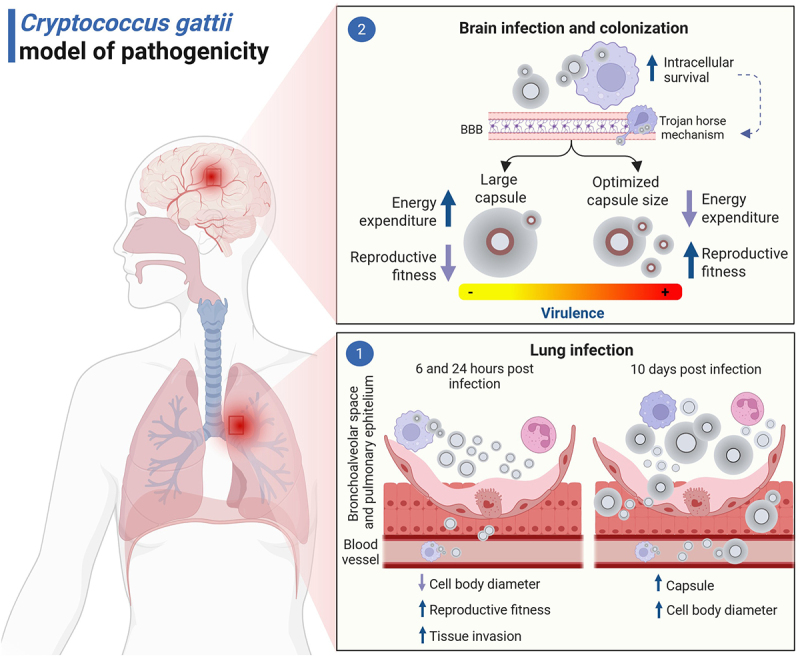
*Cryptococcus gattii* proposed pathogenicity model. Morphological changes play an essential role in the establishment of *C. gattii* infection [1]. Initially, the reduction in cell body size contributes to an increased reproductive fitness and tissue invasion ability. After 10 days of infection, all strains increased polysaccharide capsule in the lungs [2]. However, only the most virulent strains demonstrated resistance to the fungicidal activity of macrophages, which favors translocation to the central nervous system (CNS) through the “Trojan horse” mechanism. After dissemination, all strains were able to infect the CNS. However, only the most virulent strains were able to colonize the brain tissue, resulting in higher mortality. At this stage, the optimized increase in the polysaccharide capsule in the brain proved to be a key factor for the virulence of *C. gattii*, by providing lower energy expenditure, favoring the multiplication in the tissue. Despite the contribution of melanin to resist the host defenses, it is not an isolated determinant of fungal burden or severity of infection, since all strains presented melanin in the CNS. The brown color on the surface of the cell body indicates melanization.

To assess *C. gattii* colonization in brain tissue and mitigate pulmonary dissemination over time, an intracranial fungal infection model was used. This model also demonstrated a higher fungal burden and proliferation rate in the CNS 10 d.p.i, for the most virulent strains (Figure 5(B,C)). In this scenario, WM779 and R265 presented smaller total cell diameter and relative capsule size compared to the less virulent strains (WM179 and WM161) (Figure 5(D–H)) (*p* < 0.0001). Cell body size was similar for all strains (Figure 5(E)). However, the most virulent strains presented an average relative capsule size between 1.0–2.0 (Figure 5(G)), which may characterize an optimized capsule size, sufficient to resist the immune response and to allow higher growth rate in the tissue. On the other hand, we observed predominantly a relative capsule size >2 (Figure 5(G)) for the less virulent strains (WM179 and WM161), which may imply greater energy expenditure for the fungal cell, affecting its maintenance in the tissue. Despite differences in virulence profiles and replication rates in the CNS, all strains were able to produce melanin in the brain tissue after intracranial infection (Figure 5(I)). This was evidenced by histopathological analysis of brain sections stained with Fontana-Masson, a silver-based technique specific for melanin detection, performed 10 days after intracranial infection. Melanin was observed as dark granular deposits in the fungal cell wall, indicating active melanization in all strains. Based on the results of this study, Figure 6 presents a model for the pathogenic mechanisms in *C. gattii* infection.

## Discusion

The results from this study revealed significant differences in the virulence of five *C. gattii* species complex strains, encompassing the different molecular types (VGI, VGII, VGIII and VGIV). Based on the average lethality and behavioral changes in a murine model, the strains were classified as hypervirulent (WM779 VGIV), virulent (R265 VGII), hypovirulent (WM178 VGII and WM179 VGI) and non-lethal (WM161 VGIII). Through *in vitro* and *in vivo* approaches, we sought to identify the determining factors for these different virulence profiles.

Initially, we observed that melanin synthesis, laccase activity, and relative capsule size *in vitro* were not associated with the strain ability to cause fatal disease in mice. Since laboratory conditions do not fully replicate host environment dynamics, *in vitro* analyses appear less predictive for characterizing *Cryptococcus* spp. virulence. This observation aligns with findings from our previous study on *C. neoformans*. Similarly, a global study of 40 *C. gattii* strains found no direct correlation between *in vitro* virulence and lethality in an invertebrate model of cryptococcosis [35]. Regarding growth performance, the non-lethal strain WM161 consistently exhibited slower proliferation rate in both YPD and MM, suggesting a lower metabolic efficiency that may influence its adaptation to different environments, including the host niche. These findings reinforce that *Cryptococcus* spp. virulence is shaped by complexes factors during interaction with the host, independent of species or genotype.

During infection, striking morphological changes were observed in the alveolar space and pulmonary epithelium. At early stages (6 h), all strains exhibited a predominance of small cells in both compartments, a trait also reported for *C. neoformans* [7]. These results suggest that small cells are a conserved characteristic among complexes, playing a crucial role during the initial interaction between *Cryptococcus* and the host. Due to their greater surface-to-volume ratio, these cells have an increased exchange of nutrients with the extracellular environment and enhanced reproductive fitness. Additionally, small cells can more easily penetrate host barriers, enhancing the infection of other tissues. This hypothesis is particularly relevant when considering the early detection of all strains in the CNS following intratracheal infection [7].

After 10 days, the polysaccharide capsule enlargement became evident, with *C. gattii* consistently showing larger capsules than *C. neoformans*. This suggests that species-specific metabolic differences, particularly in the utilization of nitrogen and carbon sources [36–38], may directly impact capsule development during infection. Indeed, previous studies have shown that *C. gattii* exhibits larger capsule in the presence of urea compared to *C. neoformans* [39]. This reinforces that metabolic ability contributes to the distinct virulence strategies between these species.

However, capsule size alone did not correlate with virulence, in contrast to the previous findings for *C. neoformans*. This observation suggests that capsular architecture appears to play a more decisive role in *C. gattii* pathogenesis. In line with this idea, Urai et al. (2015) compared strains of *C. gattii* and *C. neoformans* with similar capsule dimensions, demonstrating that the deacetylation of glucuronoxylomannano (GXM) in the capsule, which was only observed in *C. gattii*, plays a crucial role in its virulence [40]. Moreover, it is possible that the polysaccharide capsule acts as a dynamic interface with the host, incorporating minor and transient components such as heat shock proteins, glucans, and chitooligomers [41–44]. These additional molecules could diversify the patterns of immune recognition, shaping host – pathogen interactions in a lineage-dependent manner. Notably, evidence that an increased distribution of chitooligomers on the surface of *C. gattii in vivo* correlates with higher brain infection rates [41] reinforces that subtle architectural differences in the capsule may significantly impact the outcome of infection.

All *C. gattii* genotypes (VGI, VGII, VGIII and VGIV) were detected in the CNS after mice lung infection. Although meningitis caused by *C. gattii* is typically linked to strains of the VGI genotype [1], cases involving the VGII and VGIV genotypes have also been reported in countries such as the China, Brazil, Mexico, India, Kuwait, and certain regions of Africa [45–50]. Furthermore, in sub-Saharan Africa, the VGIV genotype is among the most prevalent in cases of cryptococcosis [51]. This highlights the virulence diversity of *C. gattii*, demonstrating that less common genotypes can also lead to severe forms of the disease.

Although all strains infected the CNS, only the strains WM779 (VGIV) and R265 (VGII) were able to increase the fungal burden in the brain tissue, indicating that severity depends not only on CNS invasion but also on the ability to persist and replicate within this niche. Two complementary mechanisms may explain this profile: (i) continuous transmigration to the CNS, potentially favored by survival within macrophages (Trojan horse strategy) and (ii) increased replication capacity once in brain tissue. The first is supported by the observation that hypervirulent strains exhibited increased survival within macrophages, indicating that the “Trojan horse” pathway may play a crucial role in this transmigration [52–54]. According to previous evidence, during lung infection, the WM779 strain presents alterations in surface molecules that facilitate recognition by macrophages in the lungs [41]. By resisting the fungicidal activity of macrophages in the respiratory tract, the fungus can migrate more easily to the CNS, resulting in an increase in the fungal burden in brain tissue [41]. The second hypothesis points to the importance of fungal multiplication rate within the brain. To investigate this, we performed intracranial infections and assessed fungal burden at early and late time points of infection. Notably, the more virulent strains demonstrated a higher replication rate in the CNS. Thus, our findings support the notion that in addition to infecting the CNS, *C. gattii* must be able to colonize brain tissue to produce more severe clinical outcomes.

During CNS colonization, *Cryptococcus* spp. can use inositol as an alternative carbon source and metabolize catecholamines during melanogenesis [55–57]. Due to its antioxidant and antiphagocytic properties, melanin synthesis has been extensively associated with fungal virulence and tropism for the CNS [58]. Interestingly, in this study, melanin synthesis in the CNS was not linked to disease severity, since both hypervirulent and non-lethal strains produced melanin during brain infection, a profile also observed for *C. neoformans in vivo*. In line with our findings, a recent multicenter study that analyzed 32 *C. gattii* isolates did not identify a direct association between *in vitro* melanin synthesis and fungal virulence [59]. These findings suggest that, although melanin contributes to resistance to the host antimicrobial arsenal, it is not an isolated determinant of fungal burden or severity of *C. gattii* infection in the CNS.

Due to its antigenic and antioxidant properties, the polysaccharide capsule may also contribute to fungal survival in the CNS [60,61]. In the current study, the most virulent strains exhibited a predominant cell population in the CNS with a relative capsule size ranging from 1.0 to 2.0, indicating that capsule thickness is relatively proportional to the cell body. Since capsule maintenance requires high energy expenditure [7], this organization may provide a more favorable balance between immune evasion and energy conservation, thereby optimizing essential metabolic processes such as proliferation. In contrast, hypovirulent strains produced disproportionately large capsules (> 2.0), likely incurring high energetic costs that impaired cell multiplication and culminated in more chronic, less lethal infections. Collectively, these findings offer new insights into the infectious biology of *C. gattii* in the CNS, although further analyses are needed to clarify the molecular mechanisms sustaining fungal survival in this niche. This question becomes even more intriguing considering that transcriptional regulators known to drive brain infection in *C. neoformans* are not functionally conserved in the *C. gattii* species complex [8], suggesting that alternative, as yet unidentified, pathways may be related to its neurotropism.

Overall, our data reinforce that *C. gattii* pathogenicity is multifactorial and shaped by dynamic strategies that evolve during infection. Early adaptation favors survival, reproduction, and dissemination, while intracellular persistence and optimized capsule regulation are critical for CNS colonization and severe outcomes. Although the use of a murine model limited the number of strains tested, this study paves the way for the investigation of the molecular mechanisms underlying the ability of *C. gattii* to survive in the central nervous system and promote distinct virulence profiles.

## Supplementary Material

Supplemental Material

## Data Availability

The data supporting the findings of this study are available within the article and are openly available in “science data bank” at https://www.scidb.cn/en/detail?dataSetId=a3d738047faf43dca094789cdf638248 [62], reference number 10.57760/sciencedb.27753.
