# Supplementary material for: Colonization of the central nervous system as a key factor in the Cryptococcus gattii pathogenicity
Source: Virulence. 2025 Nov 24;16(1):2594289. doi: 10.1080/21505594.2025.2594289 (PMC12667668; doi:10.1080/21505594.2025.2594289)
Supplement: Supplemental Material [file KVIR_A_2594289_SM0744.docx]

Table S1. SHIRPA (evaluated parameters)

| Functional categories | Parameters (7, 24, 25) |
| --- | --- |
| Muscle tone and strength | Grip strength, body tone, limb tone, abdominal tone |
| Motor behavior | Body position, tremor, locomotor activity, pelvic elevation, gait, tail elevation, trunk curl, limb grasping, wire maneuver, negative geotaxis |
| Neuropsychiatric state | Spontaneous activity, transfer arousal, touch escape, positional passivity, biting, fear, irritability, aggression, vocals |
| Autonomous function | Respiration rate, defecation, urination, palpebral closure, piloerection, skin color, heart rate, lacrimation, salivation |
| Reflex and sensory function | Startle response, visual placing, pinna reflex, corneal reflex, toe pinch, righting reflex |
